# Feature Selection Methods for Zero-Shot Learning of Neural Activity

**DOI:** 10.3389/fninf.2017.00041

**Published:** 2017-06-23

**Authors:** Carlos A. Caceres, Matthew J. Roos, Kyle M. Rupp, Griffin Milsap, Nathan E. Crone, Michael E. Wolmetz, Christopher R. Ratto

**Affiliations:** ^1^Applied Physics Laboratory, Johns Hopkins UniversityLaurel, MD, United States; ^2^Department of Biomedical Engineering, Johns Hopkins UniversityBaltimore, MD, United States; ^3^Department of Neurology, Johns Hopkins MedicineBaltimore, MD, United States

**Keywords:** zero-shot learning, transfer learning, semantics, fMRI, electrocorticography, feature selection, BCI

## Abstract

Dimensionality poses a serious challenge when making predictions from human neuroimaging data. Across imaging modalities, large pools of potential neural features (e.g., responses from particular voxels, electrodes, and temporal windows) have to be related to typically limited sets of stimuli and samples. In recent years, zero-shot prediction models have been introduced for mapping between neural signals and semantic attributes, which allows for classification of stimulus classes not explicitly included in the training set. While choices about feature selection can have a substantial impact when closed-set accuracy, open-set robustness, and runtime are competing design objectives, no systematic study of feature selection for these models has been reported. Instead, a relatively straightforward feature stability approach has been adopted and successfully applied across models and imaging modalities. To characterize the tradeoffs in feature selection for zero-shot learning, we compared correlation-based stability to several other feature selection techniques on comparable data sets from two distinct imaging modalities: functional Magnetic Resonance Imaging and Electrocorticography. While most of the feature selection methods resulted in similar zero-shot prediction accuracies and spatial/spectral patterns of selected features, there was one exception; A novel feature/attribute correlation approach was able to achieve those accuracies with far fewer features, suggesting the potential for simpler prediction models that yield high zero-shot classification accuracy.

## 1. Introduction

The curse of dimensionality is severe in neuroimaging data, and therefore prediction algorithms trained on neural data must take this into account to avoid overfitting. Across imaging modalities, there are often very large sets of potential neural features or dimensions, and they are often recorded across a relatively limited set of stimuli and samples. In functional magnetic resonance imaging (fMRI), responses from tens of thousands of voxels (or more) are commonly analyzed over multiple time points. Magnetoencephalography (MEG), electroencephalography (EEG), and electrocorticography (ECoG) involve only several hundred channels at most, but when combined with high sampling rates and rapidly varying neural responses, the resulting dimensionality is often similar to fMRI. This imbalance between features and samples is a common burden in hypothesis testing and model estimation in neuroscience.

A variety of statistical corrections (Nichols, 2012), feature selection (Guyon and Elisseeff, 2003), and dimensionality reduction techniques (Mwangi et al., 2014) are typically used to address this ever-present issue. The most straightforward approach involves *a priori* hypotheses about what spatial or temporal features are likely to be informative. For example, features can be selected and/or aggregated based on atlases (Chu et al., 2012), parcellations (Desikan et al., 2006; Glasser et al., 2016; Gordon et al., 2016), temporal windows, or frequency bands (Hotson et al., 2016).

Unfortunately, for many studies and applications there are no strong *a priori* hypotheses about feature importance. One such area is neural-semantic prediction, which is used by *zero-shot* stimulus classification algorithms (Palatucci et al., 2009) for identifying classes lacking in training data. Various authors have demonstrated the ability to learn mappings between neural features and semantic attributes, mostly in fMRI studies (Mitchell et al., 2008; Palatucci et al., 2009; Pereira et al., 2011; Sudre et al., 2012; Wehbe et al., 2014), and more recently, ECoG (Rupp et al., 2017). Once the neural-semantic mapping is learned, novel stimuli can be characterized inductively using the semantic distance. Considering the large number of potential open set stimuli and the small amount of data available to train, feature selection can be a path toward generalization by ensuring that zero-shot predictors do not overfit to small data sets.

A simple approach to feature selection, termed *correlation stability*, has been successfully applied by many studies of semantic representations in the brain (Shinkareva et al., 2008; Just et al., 2010; Chang et al., 2011; Pereira et al., 2011; Levy and Bullinaria, 2012; Sudre et al., 2012; Wehbe et al., 2014; Chakrabarti et al., 2015). In this approach, neural features are ranked based on how stable their activation profiles are across repetitions of the same class of stimuli, where stability is measured via correlation. The most highly-ranked features are then chosen to train the predictive model. The logical and algorithmic simplicity, performance, and computational efficiency have made correlation stability a popular choice for feature selection in neuroimaging data sets, including those used for zero-shot learning.

Although numerous studies using correlation stability have reported positive results for zero-shot stimulus classification, no systematic comparisons with other feature selection techniques has been reported. In the current study, we compared correlation-based stability to several other feature selection techniques with particular attention to the tradeoff between prediction accuracy and the resulting model complexity, which may be of interest in brain-computer interface (BCI) engineering. Four methods were compared to correlation-based stability: a Wrapper Method, Fisher's Method, Mutual Information-Based Stability, and a novel Attribute/Feature Correlation technique. Following previous uses of correlation-based stability, each of these methods was used to select features for a neural *encoding model* (i.e., predicting neural feature values from semantic attributes) and a semantic *decoding model* (i.e., predicting semantic attributes from neural features values) to predict classes that were not included in the training set.

To assess how feature selection methods perform across imaging modalities, visual stimuli were classified using two very different modalities: voxel activity from whole-brain fMRI data published by Mitchell et al. (2008), and spectral-temporal features across subdural electrodes collected by our group from ECoG patients performing object-naming with the same stimuli (Rupp et al., 2017). Results show that a novel attribute/feature correlation technique is an improvement over standard correlation stability, by which maintaining high performance while substantially reducing the number of features required to achieve it. Further analysis seems to indicate that this improvement might be the result of a more diverse spatial distribution (in fMRI) or temporal distribution (in ECoG) of the chosen features.

## 2. Materials and methods

### 2.1. Zero-shot transfer learning models

#### 2.1.1. Overview

Zero-shot stimulus classification can be implemented through encoding and decoding models which decompose of a class of stimuli into its constituent attributes or features (i.e., visual, acoustics, phonological, spatial, or semantic attributes). Using this approach, models can be learned for relating a set of attributes to the neural responses evoked by various classes of stimuli. This process recasts the classification problem as a transfer learning problem. This type of computational model has been used extensively for the study of visual and semantic feature representation in the brain, as well as other applications such as computer vision (Burlina et al., 2015) and landmine detection (Colwell and Collins, 2016).

In practice, zero-shot transfer learning involves the mapping between (semantic) attributes (**x** ∈ ℝ^*P*^) and (neural) features (**y** ∈ ℝ^*M*^). Encoding consists of the mapping **x** ↦ **y**, and decoding corresponds to **y** ↦ **x**. Zero-shot prediction is performed by a distance-based classifier in the map output space. Since neural data is inherently high-dimensional, it is assumed that *M*≫*P*, so feature selection only needs to be performed on **y**.

#### 2.1.2. Encoding model

Encoding takes the form of a linear regression of **x** onto each *y*_*m*_, for *m* = 1, 2, …, *M*:

(1) $$\begin{array}{r} {ŷ}_{m}={\text{x}}^{T}{\text{w}}_{m}^{\left(en\right)} \end{array}$$

The parameter vector ${\text{w}}_{m}^{\left(en\right)}$ consists of the regression weights for encoding the *m*th feature, and is learned from training data. Therefore, *M* individual encoding regression models are learned. In this work, ridge regression was used as a coefficient shrinkage method to safeguard against overfitting (Hastie et al., 2001). The optimal encoding weights are given by

(2) $$\begin{array}{r} {\text{w}}_{m}^{\left(en\right)}=\underset{\text{w}}{\mathrm{argmin}}||\text{X}\text{w}-{\text{y}}_{m}|{|}_{2}^{2}+\lambda^{\left(en\right)}||\text{w}|{|}_{2}^{2}\\ ={\left({\text{X}}^{T}\text{X}+\lambda^{\left(en\right)}\text{I}\right)}^{-1}{\text{X}}^{T}{\text{y}}_{m}, \end{array}$$

where **X** is the *T* × *P* matrix of semantic attributes (each row normalized to unit length), where *T* is the total number of training samples, **y**_*m*_ is the *T* × 1 vector of values of the *m*th neural feature (normalized to zero mean, unit variance), and λ^(*en*)^ is a regularization parameter set experimentally.

#### 2.1.3. Decoding model

The decoding model is a reversal of the encoding model, in which each attribute is predicted independently from the set of features. Using the above notation, decoding takes the form of a linear regression of **y** onto each *x*_*p*_, for *p* = 1, 2, …, *P*:

(3) $$\begin{array}{r} {\hat{x}}_{p}={\text{y}}^{T}{\text{w}}_{p}^{\left(de\right)} \end{array}$$

The parameter vector ${\text{w}}_{p}^{\left(de\right)}$ consists of the regression weights for decoding the *p*th attribute, which are learned from training data. Therefore, *P* individual decoding regression models are learned. The ridge regression solution for the optimal decoding weights is given by

(4) $$\begin{array}{r} {\text{w}}_{p}^{\left(de\right)}=\underset{\text{w}}{\mathrm{argmin}}\left\{||\text{Y}\text{w}-{\text{x}}_{p}|{|}_{2}^{2}+\lambda^{\left(de\right)}||\text{w}|{|}_{2}^{2}\right\}\\ ={\left({\text{Y}}^{T}\text{Y}+\lambda^{\left(de\right)}\text{I}\right)}^{-1}{\text{Y}}^{T}{\text{x}}_{p}, \end{array}$$

where **Y** is the *T* × *M* matrix of features (each column normalized to zero mean, unit variance), **x**_*p*_ is the *T* × 1 vector of values of the *p*th attribute (normalized to unit length), and λ^(*de*)^ is the regularization parameter.

#### 2.1.4. Zero-shot stimulus prediction

After using regression to transfer between features and attributes, zero-shot stimulus prediction can be performed by a distance-based classifier. In this work, the cosine distance was used used so that differences in magnitude between the predicted and actual vectors are ignored, and only relative differences between the vector elements are taken into account (Palatucci et al., 2009; Jelodar et al., 2010).

Let the cosine distances resulting from the encoder output be denoted as

(5) $$\begin{array}{r} d_{\phi }^{\left(en\right)}=\frac{\hat{\text{y}}\cdot {\text{y}}_{\phi }}{\left||\hat{\text{y}}||\;||{\text{y}}_{\phi }|\right|}, \end{array}$$

where **y**_ϕ_ is the average feature vector for stimulus ϕ. Therefore, predicting the class (neural activation) ϕ via encoding takes the form of

(6) $$\begin{array}{r} {\hat{\phi }}^{\left(en\right)}=\underset{\phi }{\mathrm{argmin}}\left\{d_{\phi }^{\left(en\right)}\right\}. \end{array}$$

Similarly, prediction via decoding takes the form of

(7) $$\begin{array}{r} d_{\phi }^{\left(de\right)}=\frac{\hat{\text{x}}\cdot {\text{x}}_{\phi }}{\left||\hat{\text{x}}||\;||{\text{x}}_{\phi }|\right|}, \end{array}$$

(8) $$\begin{array}{r} {\hat{\phi }}^{\left(de\right)}=\underset{\phi }{\mathrm{argmin}}\left\{d_{\phi }^{\left(de\right)}\right\}, \end{array}$$

where **x**_ϕ_ is the vector of true attribute values for stimulus ϕ.

### 2.2. Data collection

#### 2.2.1. Neural stimuli and semantic attributes

**Figure 1:**
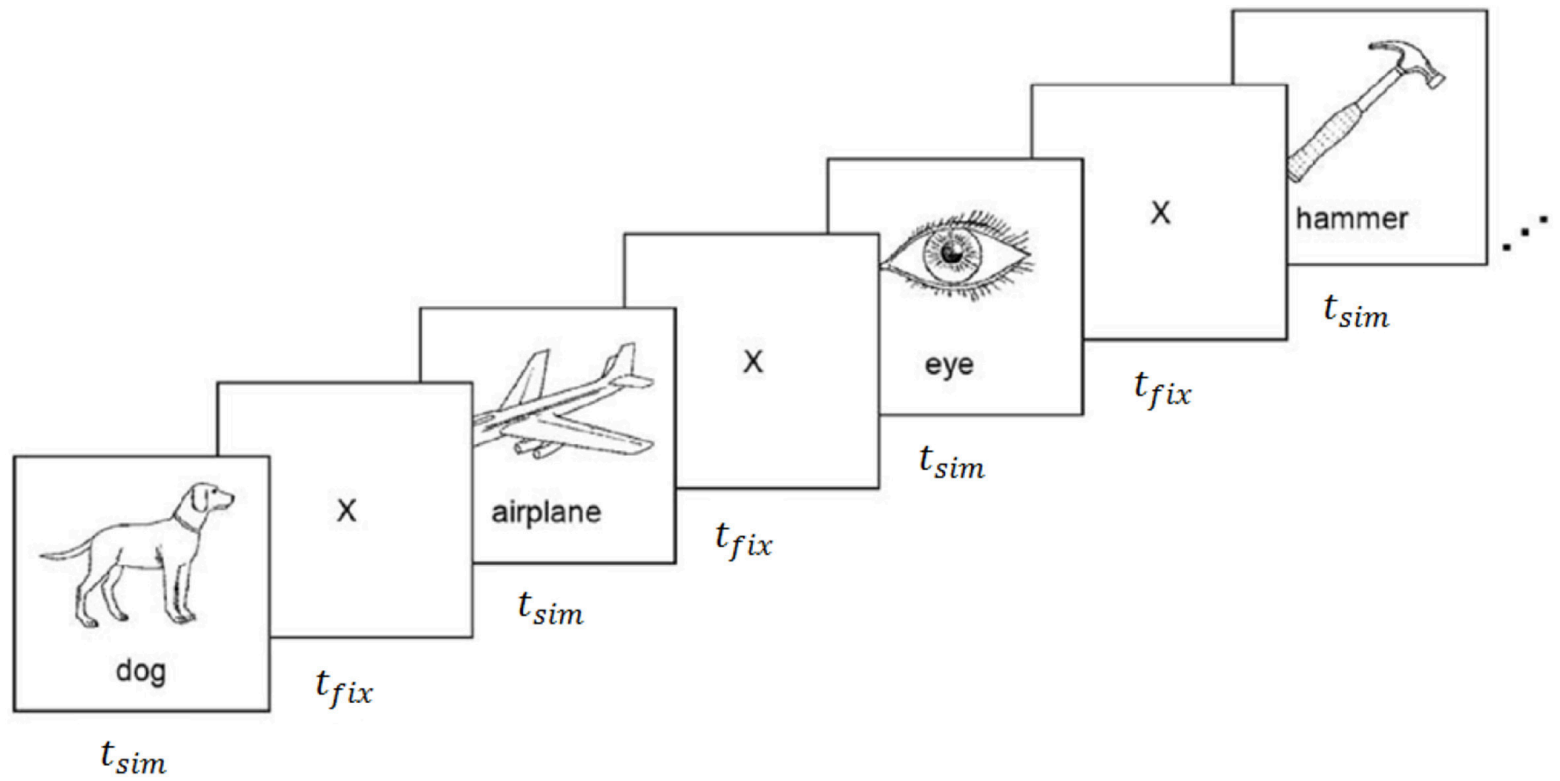
fMRI and ECoG data collection procedure. Stimuli were presented for *t*_*stim*_ seconds followed by a fixation cross for *t*_*fix*_ seconds. Images were composed of black outlines on white backgrounds for fMRI experiments, and white outlines on black backgrounds for ECoG experiments. Reproduced from Mitchell et al. (2008) with permission from The American Association for the Advancement of Science.

The experiments carried out for this work utilized fMRI data collected during a property-contemplation task originally reported by Mitchell et al. (2008), and ECoG data collected during a similar task using the same stimuli (Rupp et al., 2017). The stimuli consisted of 60 line drawings of various animals, body parts, buildings, building parts, clothing, furniture, insects, kitchen utensils, man-made objects, tools, vegetables, and vehicles (Figure 1). Each of the 60 stimuli was uniquely mapped to a vector of *P* = 218 semantic attributes proposed by Palatucci et al. (2009). The attributes were generated by crowdsourcing answers to a series of 218 questions via Amazon Mechanical Turk. All 218 questions were asked of 1,000 different objects, including all 60 of the objects included in this study. Questions probed a variety of semantic properties, including size, usage, composition, and category, with answers on an ordinal scale from 1 to 5. The attribute vectors were rescaled to the range [−1, 1] and normalized to unit length prior to training the encoding and decoding models.

#### 2.2.2. Functional MRI

The fMRI data set used in this work was originally cataloged by Mitchell et al. (2008). Data was collected from nine college-age participants who were presented with each line drawing for *t*_*stim*_ = 3 s, followed by a fixation period of *t*_*fix*_ = 7 s. The stimulus set was randomly permuted, and shown to each participant six times. During stimulus presentations, the participants were instructed to think about the object properties. fMRI images were collected on a Siemens Allegra 3.0T scanner and include seventeen 5-mm thick oblique-axial slices imaged with a 1-mm gap between slices. The resulting images were 64 × 64 pixels in size where a pixel corresponds to a 3.125 × 3.125 × 5-mm voxel. Feature preprocessing steps included motion and timing compensation, filtering, normalization to MNI space, and resampling. Then voxel activations were calculated as the deviation from the fixation condition for each stimulus. These activation values served as the neural features (*y*_*m*_) for the fMRI experiment. A breakdown of the total number of features for each participant can be found in Table 1.

**Table 1:** Number of features per subject and collection modality.

**fMRI**		**ECoG**	
**Participant**	**No. of features**	**Participant**	**No. of features**
1	2,721	1	2,088
2	2,657	2	864
3	2,581	3	2,208
4	2,549	4	2,088
5	2,575	5	2,736
6	2,490	6	2,328
7	2,469		
8	2,501		
9	2,668		

#### 2.2.3. Electroencephalography

**Figure 2:**
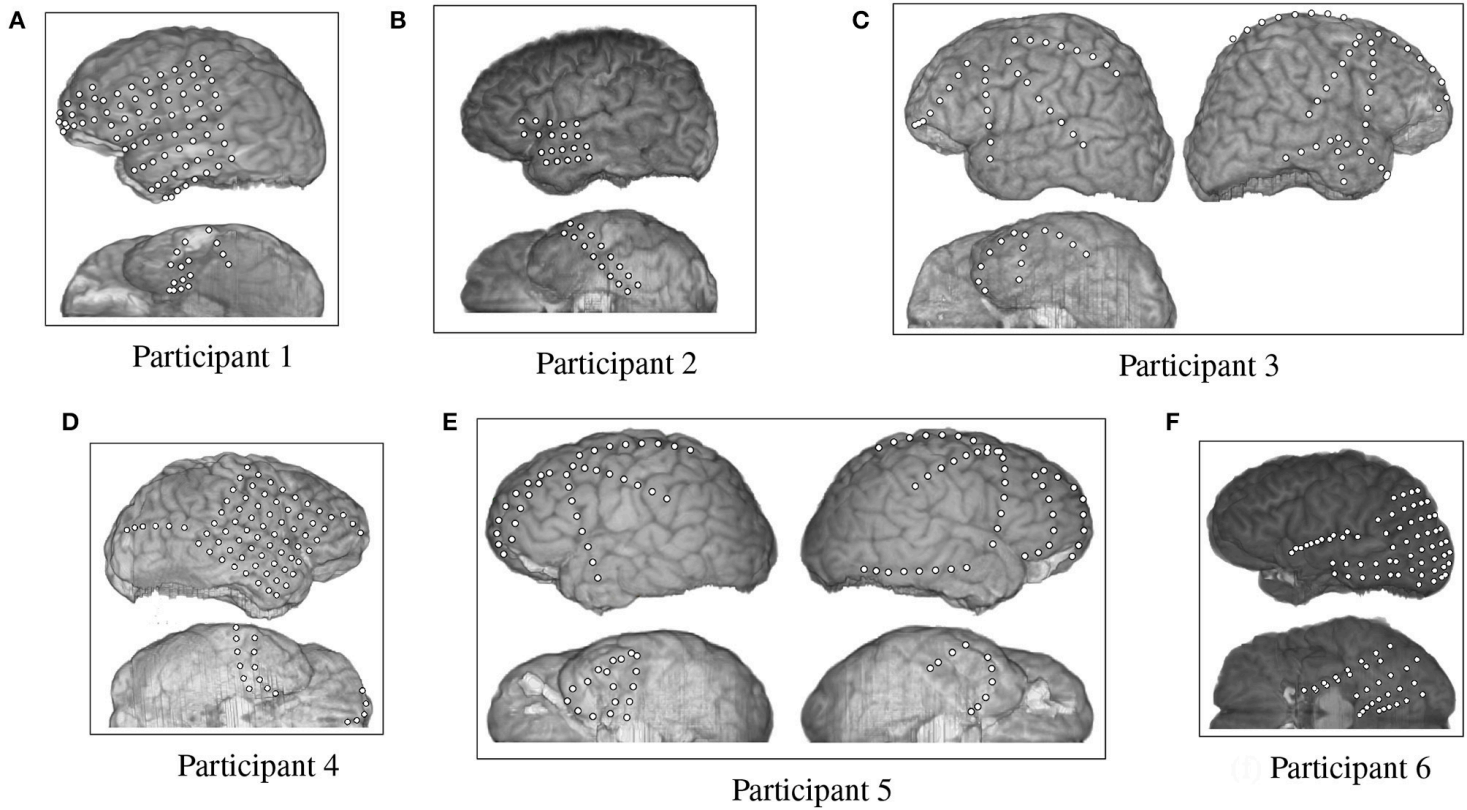
Electrode placement grid for the ECoG participants overlaid on a magnetic resonance imaging (MRI) scan for anatomical reference.

The fMRI data provided by Mitchell et al. were supplemented by an ECoG data set collected by Rupp et al. (2017) from six participants at Johns Hopkins Hospital. The test participants were undergoing epilepsy monitoring for localization of seizure focus. All participants provided informed consent, and the procedures were approved by the Institutional Review Board of Johns Hopkins Medicine. All six participants had different arrangements of ECoG grids and strips that were emplaced for clinical purposes (Figure 2). Participants 1 through 6 had 87, 36, 92, 87, 114, 97 electrodes respectively, each of which were 4 mm in diameter and spaced 10 mm apart, center-to-center.

ECoG data was recorded using a similar collection paradigm as Mitchell et al. (2008). White line drawings were presented on a black background, with a centered white fixation cross present during inter-stimulus intervals. Stimuli were shown for *t*_*stim*_ = 1 s, with a rest interval *t*_*fix*_ varying randomly between 3.5 and 4.5 s. Participants were instructed to name the image as soon as possible, or pass on images when necessary. The stimulus set consisted of the same 60 object classes as in the fMRI experiment. Six blocks of data were collected per patient, where all 60 objects were shown in pseudo-random order. Picture-naming was selected for this experiment to ensure participant compliance and to provide a means of verifying correct object identification. ECoG signals were sampled at 1,000 Hz, digitized, and recorded using the BlackRock Neuroport system. Experimental equipment, including a microphone and a photodiode, were also recorded through the analog inputs of the recording system. For participants with more than 128 electrodes, two Neuroport systems were used, with analog channels recorded separately on each recording system to aid in synchronization. The stimulus presentation and data recording were implemented with BCI2000 (Schalk et al., 2004).

After data collection, ECoG channels that were identified to contain excessive noise upon visual inspection were discarded. A common-average reference was used to spatially filter the signals, where each electrode was referenced to the grid or strip to which it belonged. Signals were then low-pass filtered with a cutoff frequency of 128 Hz to prevent aliasing, resampled to 256 Hz, and time-gated to a time range from stimulus onset to 750 ms post stimulus onset. The analysis period was restricted to minimize contamination from the participant's spoken response. The spectrogram of the time-gated ECoG data was computed using the short-time fast Fourier transform (FFT) with 500 ms time windows and 50% overlap.

**Figure 3:**
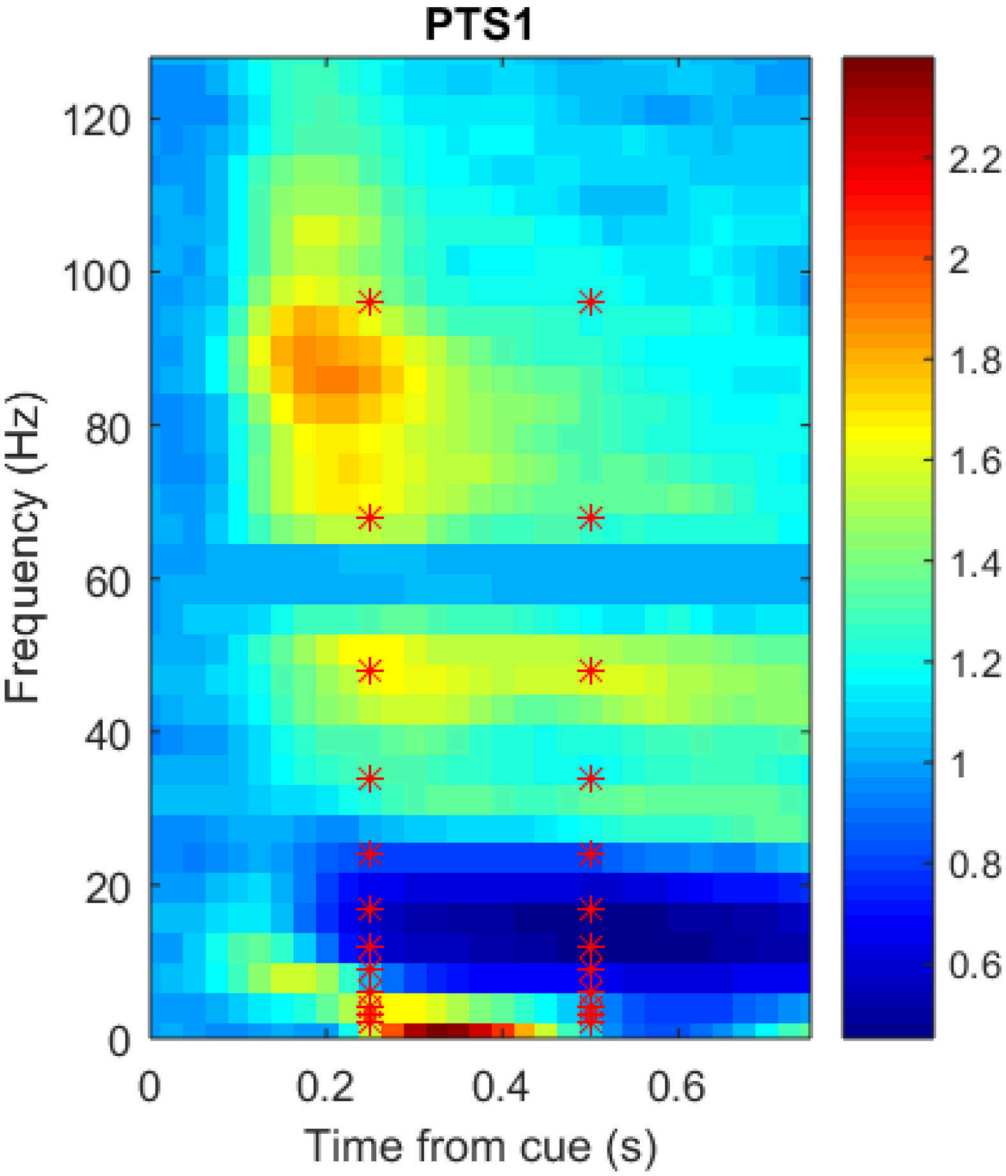
Sample spectrogram, averaged over all 360 trials, for the most postero-medial electrode on the basal temporal surface of Participant 1's ECoG data set. The horizontal axis corresponds to time in seconds (from stimulus onset), the vertical axis corresponds to frequency (Hz), and the color scale corresponds to the complex magnitude of the short-time FFT. Data from the outlined region were averaged into 24 overlapping frequency and time subregions centered at each red asterisk prior to use as neural features.

Figure 3 shows a portion of a spectrogram that demonstrates the details of specific frequency activation after stimulus onset. In this figure, the magnitude of the recorded signal has been normalized by the magnitude of a baseline signal recorded 1,000 ms prior to the stimulus. Time/frequency features were extracted from the spectrogram of each ECoG signal in 24 subregions (each indicated by a red asterisk) made up of two overlapping time windows centered at 250 and 500 milliseconds after onset, as well as 12 overlapping frequency windows. Because the number of electrodes varied per subject, the number of potential ECoG features varied as well; the number of features per participant is detailed in Table 1. The breakdown of selected features by frequency bin and location, as well as the performance of those features in zero-shot decoding and encoding, was used to evaluate various feature selection techniques.

### 2.3. Feature selection techniques

#### 2.3.1. Overview

Feature selection methods are generally organized into two categories, *wrapper methods* and *filter methods* (Guyon and Elisseeff, 2003). Filter methods work by applying a ranking criterion to each feature, independent of the regression model, and the top-ranked features are kept in the final model design. Wrapper methods, on the other hand, use a regression model to continuously test combinations of features while keeping track of the best possible combination. Although wrapper methods offer a more systematic search of the feature space, they also require long computation times. A mix of filter and wrapper methods were considered in this study. To asses their ability to accommodate zero-shot learning, leave-one-class-out (LOCO) cross-validation was used to validate all of the feature selection techniques. Details regarding their implementation are summarized in the following subsections.

#### 2.3.2. Correlation-based stability

Correlation-based ranking was originally proposed as a method for ranking and down-selecting fMRI voxels according to their stability, as measured by pair-wise correlation across blocks of repeated trials (Shinkareva et al., 2008). Since then, many studies have relied upon correlation-based stability selection prior to training an encoding or decoding model (Mitchell et al., 2008; Palatucci et al., 2009; Chang et al., 2011; Pereira et al., 2011, 2013; Levy and Bullinaria, 2012; Chakrabarti et al., 2015). The motivation for selecting features based on stability lies in the expectation that semantic information will be encoded in a repeatable manner. It is implicitly assumed that any drift in information-bearing neural signals between trials is linear.

The correlation stability measure is calculated for each feature, where the correlation is measured by class and within pairs of trial blocks and then averaged over all possible block pairings. The features are then ranked according to average correlation, in descending order. The *M* features with the largest average correlation, i.e., the *M* most stable features across blocks, are selected for use in the encoding and decoding models.

#### 2.3.3. Mutual information based stability

The correlation measure can accommodate a linear drift of neural feature values between blocks. A more general measure that can accommodate nonlinear relationships is *mutual information*. The mutual information between two random variables *Y*_1_ and *Y*_2_ is the amount by which uncertainty in *Y*_1_ is reduced by knowing *Y*_2_ (and vice versa) (Cover and Thomas, 2006). Mutual information is computed by

(9) $$\begin{array}{r} I\left(Y_{1};Y_{2}\right)=\int \int p\left(y_{1},y_{2}\right)\log\frac{p\left(y_{1},y_{2}\right)}{p\left(y_{1}\right)p\left(y_{2}\right)}dy_{1}dy_{2}, \end{array}$$

where *p*(*y*_1_) and *p*(*y*_2_) are the marginal probability density functions (PDFs) of *Y*_1_ and *Y*_2_, and *p*(*y*_1_, *y*_2_) is the joint PDF of *Y*_1_ and *Y*_2_. It is assumed here that *Y*_1_ and *Y*_2_ are Gaussian-distributed. Therefore, the mutual information between two neural features is computed by

(10) $$\begin{array}{r} I\left(Y_{1};Y_{2}\right)=\frac{1}{2}\log\left(\frac{\sigma_{1}^{2}\sigma_{2}^{2}}{\left|\Sigma \right|}\right), \end{array}$$

where $\sigma_{1}^{2}$ and $\sigma_{2}^{2}$ are the estimated variances of *Y*_1_ and *Y*_2_, respectively, and Σ is their estimated covariance matrix.

The process of computing average mutual information for each feature and selecting *M* features for the models is identical to that described for correlation stability with the obvious exception that mutual information is computed rather than correlation. The marginal and joint distributions of each pair of blocks are estimated using maximum likelihood estimates of the sample mean and covariance.

#### 2.3.4. Attribute/feature correlation

This method supplements the basic correlation stability method with a prior step that utilizes the attributes. First, the neural feature with the highest correlation (over all trials and classes) to each of the attributes is found. This process reduces the pool of candidate features to a small number *M*′ ≤ *P. M*′ may be less than *P* because some features may be the most highly-correlated with multiple attributes. This subset of neural features are then ranked using the standard correlation-based stability method.

#### 2.3.5. Fisher's method

Fisher's method analyzes each feature's distribution through their mean and standard deviation, where each feature is rated based on its spread from the mean for each class (Duda et al., 2001; Guyon and Elisseeff, 2003). The Fisher score for the *m*th feature, *S*_*F*_(*Y*_*m*_), is computed as

(11) $$\begin{array}{r} S_{F}\left(Y_{m}\right)=\frac{\sum_{i=1}^{N}n_{i}{\left(\mu_{i,m}-\mu_{m}\right)}^{2}}{\sum_{i=1}^{N}n_{i}\sigma_{i,m}^{2}}, \end{array}$$

where *N* is the number of classes, *n*_*i*_ is the number of trials using the *i*th class as stimulus, μ_*i,m*_ is the sample mean of *Y*_*m*_ conditioned on the *i*th class, μ_*m*_ is the sample mean of *Y*_*m*_ over all trials and classes, and $\sigma_{i,m}^{2}$ is the sample variance of *Y*_*m*_ conditioned on the *i*th class. According to Equation 11, features which cluster more tightly around their mean value per class are given higher scores. Scores are sorted in descending order and the *M* features with the highest score are chosen.

#### 2.3.6. Ridge regression wrapper

Finally, a wrapper method based upon the ridge regression model was implemented as follows:

1. One hold-out class is removed from the set of all classes. (This is not to be confused with the LOCO cross-validation class. In the work here, we start with 59 classes in step 1 and steps 2–3 below operate on 58 classes).
2. The encoding model is trained on the remaining classes using Equation 2 to obtain ${\text{w}}_{m}^{\left(en\right)}$ for *m* = 1, 2, …, *M*.
3. The semantic vector **x** corresponding to the held-out class is used to predict the neural features **y**_*m*_ (*m* = 1, 2, …, *M*), resulting from that stimulus using Equation 1.
4. Repeat steps 1-3 for all classes.
5. The correlation is calculated between each of the predicted features ${\hat{\text{y}}}_{m}$ and the average true **y**_*m*_ for the held-out class.
6. Select the *M* features with the highest average correlations.

## 3. Results

### 3.1. Overview

Using the feature selection techniques described in Section 2.3, experiments were conducted to assess performance of zero-shot classification via semantic encoding/decoding in fMRI and ECoG. The zero-shot problem was simulated by employing LOCO cross-validation; feature selection and training was performed using 59 of the 60 classes, and one class was held out for testing. Therefore, the number of trials used to train the models was *T* = 6 × 59 = 354 per subject. The efficacy of the feature selection techniques were compared in terms of prediction accuracy as well as in the locations (in the case of fMRI) or the frequencies (in the case of ECoG) of the features that were selected.

### 3.2. Analysis of zero-shot classification accuracy

The performance of zero-shot classification is measured via *mean rank accuracy* (MRA). The MRA represents the average rank accuracy (RA) of the zero-shot test class, taken across the full set of 60 classes ranked according to the cosine distance. RA is the relative (%) rank position of the test class ϕ within a ranked list of predicted class:

(12) $$\begin{array}{r} RA_{\phi }=100\times \left(\frac{60-r_{\phi }}{59}\right) \end{array}$$

where *r*_ϕ_ is the rank of the distance to class ϕ computed using Equation (6) or (8). MRA is computed by averaging RA over the 60 LOCO folds:

(13) $$\begin{array}{r} MRA=\frac{1}{60}\overset{60}{\underset{\phi =1}{\sum }}RA_{\phi } \end{array}$$

For both encoding and decoding, MRA was calculated separately for each participant using each of the feature selection methods. Furthermore, the MRA was also calculated in a cumulative manner as increasing numbers of neural features (*M*) were incorporated into learning the encoding and decoding models, up to a maximum of 500 features. The order in which neural features were incorporated was based on the score produced by each feature selection method. Five values of λ^(*de*)^ (logarithmically-spaced between 1 and 10) and λ^(*en*)^ (logarithmically-spaced between 100 and 1,000) were tested for regularization.

**Figure 4:**
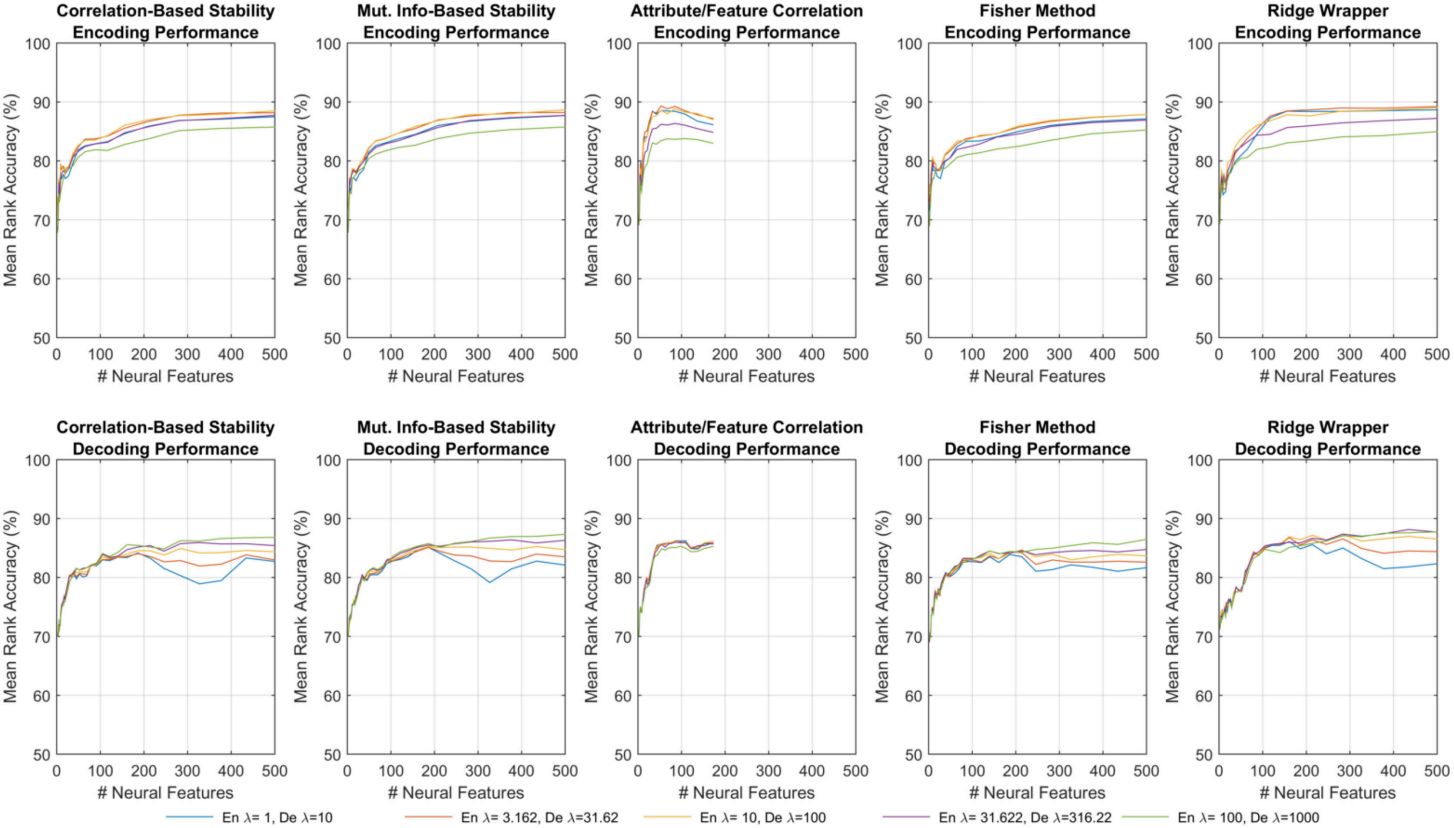
Results of encoding **(Top row)** and decoding **(Bottom row)** for the first fMRI participant. Each sub-panel shows MRA vs. the number of neural features for a specific feature selection method, where each trace corresponds to a different λ^(*en*)^ or λ^(*de*)^.

Figure 4 shows one such set of results for the first fMRI participant. Each subplot shows the MRA as a function of the number of neural features. For all but one of the feature selectors, the number of neural features was limited *M* ≤ 500. For the Attribute/Feature Correlation method, the number of features is practically limited to *M* ≤ *P* = 218, but due to some neural features correlating best with multiple semantic attributes the effective limit is less. Each colored trace represents a different value of the regularization parameter, which is λ^(*en*)^ in the top row and λ^(*de*)^ in the bottom row. In encoding, a small amount of regularization is needed, with larger λ^(*en*)^ resulting in degraded performance. In contrast, with larger number of features, decoding performance is improved as λ^(*de*)^ increases. These contrasting results are consistent with expectations; because the number of possible input features is much larger in the decoding problem, regularization plays a larger role in preventing the decoding model from being overfit to the training data.

The results shown in Figure 4 represent all of the results achieved for the first fMRI participant using each feature selection method, and the observed trends were similar for the other fMRI and ECoG participants. All subsequent discussions of the results are based upon the peak MRAs achieved over all possible choices of regularization parameters. It is clear that for most feature selection methods, MRA increases sharply with the first 100–200 features, and then levels off around 500 features. However, the Attribute/Feature Correlation method allows for a similar peak MRA but with far fewer features. This effect is analyzed further in Section 3.4.

**Figure 5:**
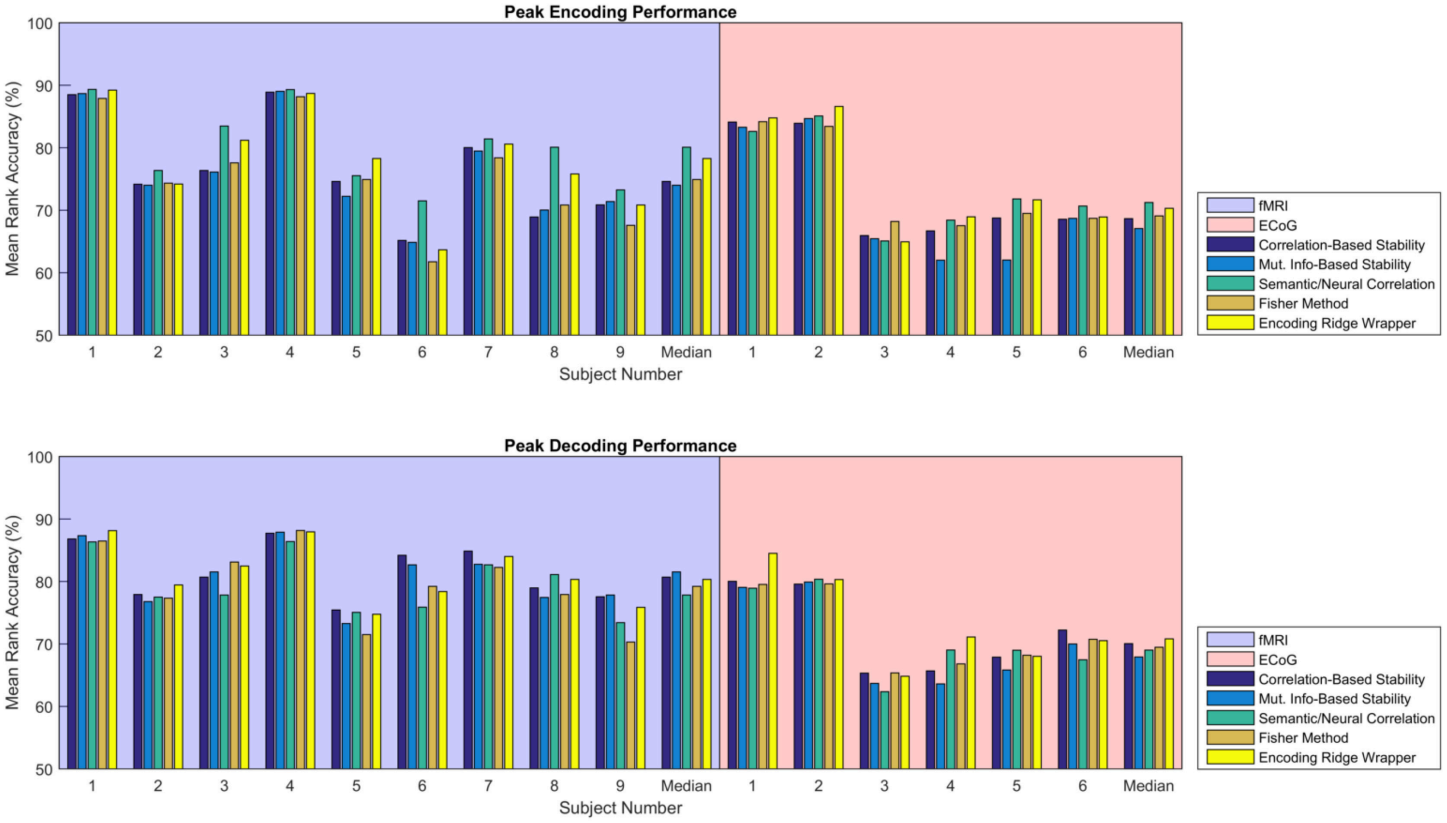
Peak MRA achieved for encoding **(Top)** and decoding **(Bottom)**. Each group of bars corresponds to a different participant, and the vertical axis represents MRA. Each colored bar represents a different feature selection method. Results are shown for the value of λ^(*en*)^ and λ^(*de*)^ that yielded the highest peak MRA.

Figure 5 shows the consolidated encoding and decoding results over all nine fMRI and six ECoG participants using the values of λ^(*en*)^ and λ^(*de*)^ that yield the highest peak MRA. The height of each bar represents the peak MRA that was achieved over all regularization parameter values and numbers of neural features. Results suggest that the best encoding/decoding performance was typically achieved using Correlation-Based Stability, Attribute/Feature Correlation, or the Ridge Wrapper. Nonetheless, the difference in performance between these methods and the Mutual Information-Based Stability and Fisher Method is usually within 5%.

### 3.3. Analysis of selected features

#### 3.3.1. Number of features to peak MRA

**Figure 6:**
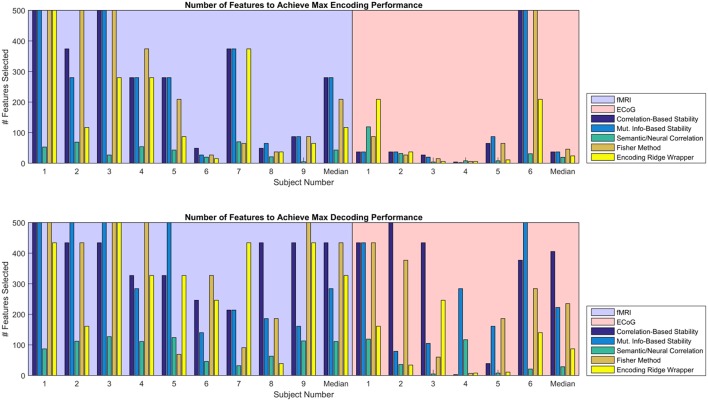
Number of features required to achieve peak MRA for encoding **(Top)** and decoding **(Bottom)**. Each group of bars corresponds to a different participant, and the vertical axis represents the number of features selected with an upper limit of 500. Each colored bar represents a different feature selection method. Results are shown for the value of λ^(*en*)^ and λ^(*de*)^ that yielded the highest peak MRA.

Given the similarity in MRA performance, the performance of the various feature selectors were also compared on the basis of the number of features required to achieve peak MRA. The number of features required for best performance is important because a simpler model is less likely to overfit the data, resulting in more robust zero-shot prediction. Figure 6 shows the number of features required to achieve peak MRA through each of the feature selection methods for all participants. The best three performing methods based on Figure 5 (Correlation-Based Stability, Attribute/Feature correlation, and the Ridge Wrapper) can be further graded on this metric. Both Correlation-Based Stability and the Ridge Wrapper required the full limit of 500 features to achieve the peak MRA. In contrast, the Attribute/Feature Correlation technique usually selected around 100 features (or substantially less), while allowing for a comparable MRA to be achieved.

#### 3.3.2. Spatial analysis of selected fMRI features

**Figure 7:**
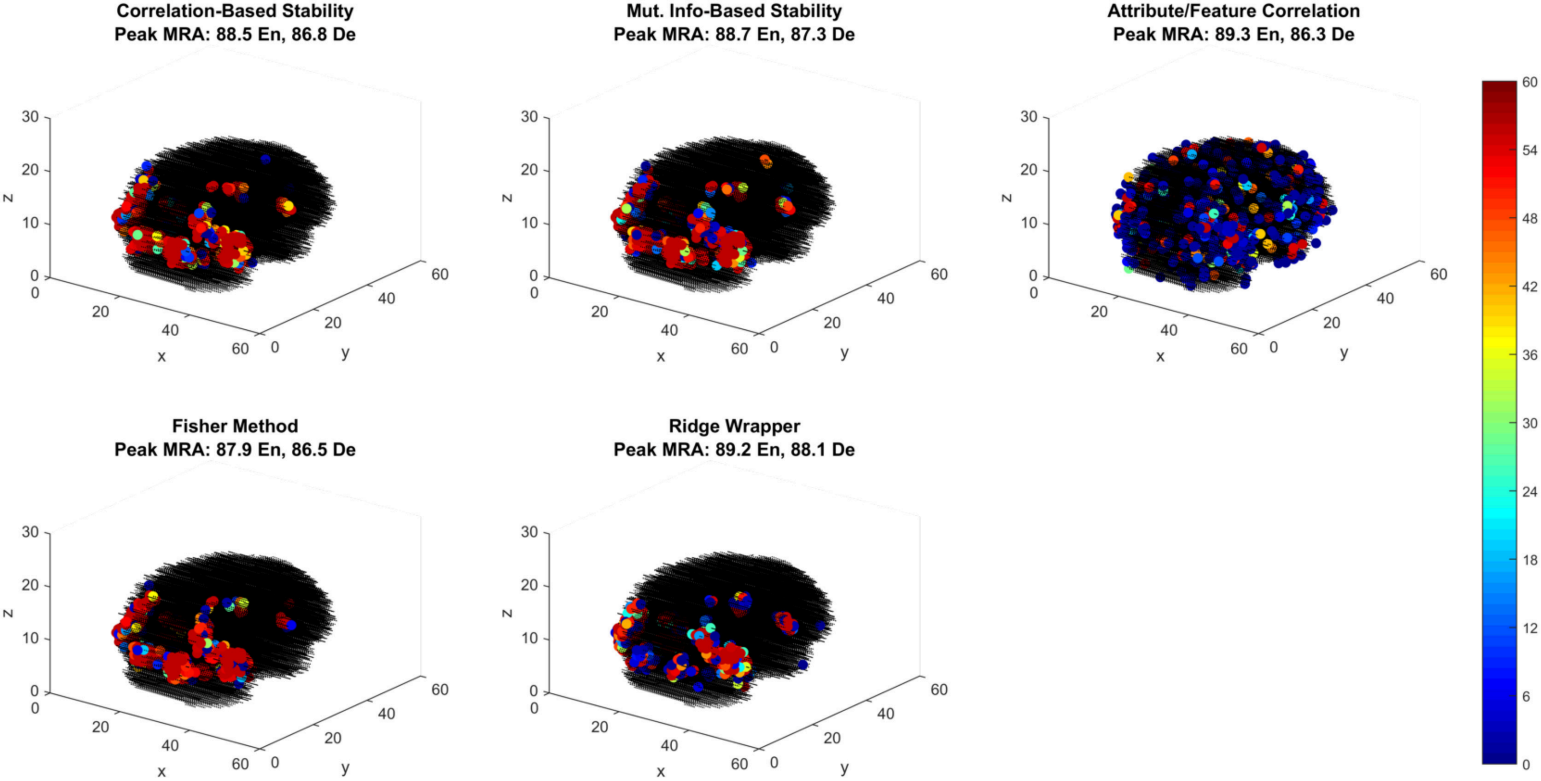
Voxel selection by method for the first fMRI participant. Each sub-panel shows the three-dimensional spatial scatter of voxel positions, with each voxel colored proportionally to the number of LOCO cross-validation folds it was selected. Each sub-panel shows the result for a different feature selection method including the peak MRA achieved in both encoding (EN) and decoding (De) modes.

Of the best performing methods, Attribute/Feature Correlation requires the least number of features. To further investigate this we explore the spatial distribution of the selected features through each selection method. While relative performance across methods is comparable, the feature selection methods that optimize with respect to classes produce one spatial distribution of features, while the feature selection method that optimizes with respect to class attributes (i.e., Attribute/Feature Correlation) produces a different distribution (Figure 7). Four of the methods selected voxels primarily along the ventral visual pathway, densely clustered in occipital and occipito-temporal cortex, with fewer voxels selected in anterior temporal, parietal and frontal cortex. This pattern of results is consistent with the literature on class perception and semantic processing associated with visual words and objects (classes) (Grill-Spector et al., 1999; Starrfelt and Gerlach, 2007; Carlson et al., 2014; Grill-Spector and Weiner, 2014; Borghesani et al., 2016).

In contrast, the Attribute/Feature Correlation method results in a set of informative voxels more distributed across cortex (and more variable across validation folds), including the anterior temporal lobe. While further analysis is required to assess the consistency of these results across patients, the pattern of results is qualitatively consistent with reports of abstract semantic representation in anterior temporal lobe and throughout cortex (Binder et al., 2009; Binder and Desai, 2011; Huth et al., 2012, 2016). Furthermore, the finding that different feature selection methods give rise to very different sets of informative voxels that are equivalent in their explanatory power suggests that choices about feature selection can have a substantial and unintended impact on the results and interpretation of studies.

#### 3.3.3. Spectral analysis of selected ECoG features

**Figure 8:**
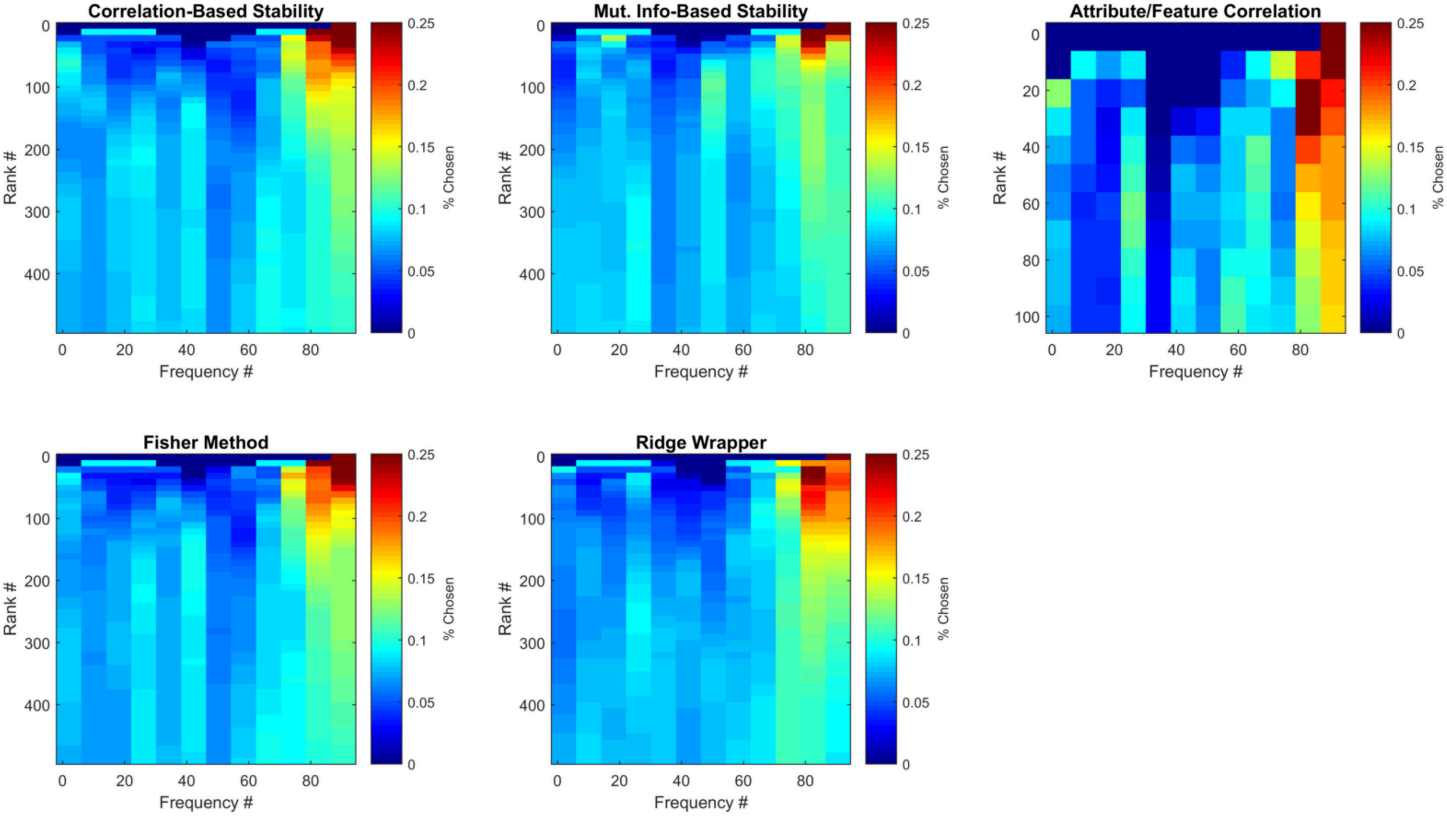
Frequencies of selected ECoG features for Subject 1. Each sub-panel is a histogram of the number of times each frequency bin was selected by a particular feature selection method. Each represents the average number of times each frequency bin was selected for this participant over all LOCO folds.

The ECoG features that were selected can be analyzed with respect to time and frequency band. Although previous work has generally prioritized high gamma (>60 Hz) frequency bands (Crone et al., 2006; Wang et al., 2011), in this work the pool of frequencies was expanded to include those in the range of ~1.4–128 Hz. Figure 8 illustrates the average number of times (over all LOCO folds) each frequency bin was selected by each method for the first participant. Results are further divided by the number of features selected from each method, where results at each feature rank are an aggregation of all previous feature ranks. These results confirm that high-gamma frequencies play an important role in capturing semantic information, as they are mainly the ones ranked first. However, selecting more features until peak MRA is achieved, quickly redistributes the chosen frequencies to a somewhat more uniform distribution, albeit with high-gamma features still more heavily represented. These results therefore suggest that frequencies outside the high-gamma band contain supplemental information that may be useful in zero-shot prediction.

### 3.4. Accuracy/complexity tradeoff

**Figure 9:**
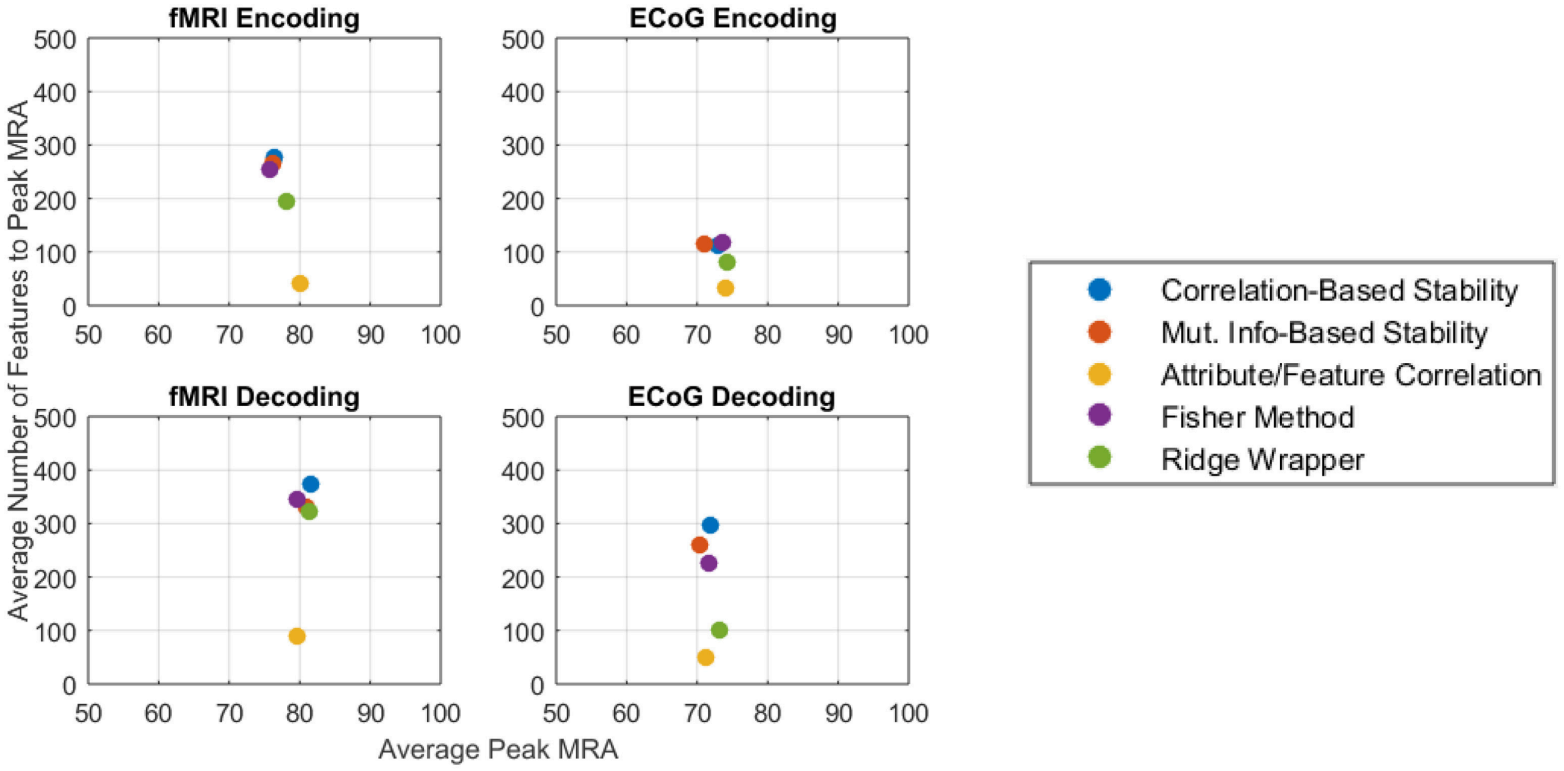
Average peak MRA vs. the number of features required for fMRI encoding **(Top-left)**, ECoG encoding **(Top-right)**, fMRI decoding **(Bottom-left)**, and ECoG decoding **(Bottom-right)**. Each colored point corresponds to a different feature selection method.

The competing objectives of maintaining high zero-shot classification performance and keeping the number of free parameters low typically presents an interesting design trade for general-purpose BCIs. However, in the ECoG and fMRI experiments presented here, no substantial trade-off exists between the peak encoding MRA, the peak decoding MRA, and the number of features required to achieve them. Figure 9 summarizes the results of this study for both fMRI (left column) and ECoG (right column) over encoding (top row) and decoding (bottom row) as an average of all patients per feature selection method. Under this representation, the best performing features selectors should have the largest possible peak MRA and the lowest possible number of features. According to these criteria, all methods have similar performance in peak MRA, while differing mostly in the number of features used. Of these methods the Attribute/Feature Correlation technique provides the result of a high MRA and the lowest possible number of features.

The Attribute/Feature Correlation technique generally requires few features to achieve a high MRA, since a few neural features may be highly correlated with many semantic attributes. The high degree of correlation allows for a linear decoding model to be fit with very small error. Since encoding accuracy is measured in terms of correlation distance, keeping the number of predicted neural features small makes the prediction more robust to regression errors. Thus, although the results of the study revealed no objective reason to select one model over another based on solely on the number of features, Occam's Razor suggest that the simpler Attribute/Feature Correlation technique, which requires less features, may be preferable for selecting features in potential BCI applications that require trading off the objectives of accuracy and complexity.

## 4. Conclusions

The necessity of feature selection is important when applying zero-shot predictive models to neuroimaging data. Furthermore, effective zero-shot learning will be necessary for BCIs to transition from the laboratory and limited clinical settings to general use. However, best practices still need to be established for algorithm development, with feature selection being a key component. This study compared the efficacy of traditional stability selection with several other feature selection techniques for encoding and decoding tasks in both fMRI and ECoG. While results did confirm that correlation-based stability can be used to achieve high prediction accuracy, the technique may select redundant information and the number of features required to achieve those performance levels can be high. However, a better engineering solution for future BCI applications may be to utilize a feature selection technique that attains similar performance, but with fewer features. The Attribute/Feature Correlation technique proposed in this study achieved that goal in both the fMRI and ECoG modalities and successfully balance the goals of simplicity and accuracy.

## Code download

The MATLAB code used to replicate the results published in this manuscript can be found at: http://www.jhuapl.edu/ott/Technologies/Copyright/Zero-Shot.asp.

## Ethics statement

This study was carried out in accordance with the recommendations of Johns Hopkins Medicine Institutional Review Board with written informed consent from all subjects. All subjects gave written informed consent in accordance with the Declaration of Helsinki. The protocol was approved by the Johns Hopkins Medicine Institutional Review Board.

## Author contributions

All authors contributed to development of the methods presented in this manuscript. Additionally, MW served as programmatic project lead, coordinating between team members from different institutions. GM and KR also conducted the data collection experiments for the ECoG modality.

### Conflict of interest statement

The authors declare that the research was conducted in the absence of any commercial or financial relationships that could be construed as a potential conflict of interest.
